# *SLC7A11*, a potential immunotherapeutic target in lung adenocarcinoma

**DOI:** 10.1038/s41598-023-45284-z

**Published:** 2023-10-25

**Authors:** Qingqing Shan, Chi Zhang, Yangke Li, Qunying Li, Yifan Zhang, Xue Li, Junqing Shi, Fengying Hu

**Affiliations:** Department of Respiration, Chengdu First People’s Hospital, No. 18, Wangxiang North Road, High-Tech Zone, Chengdu, 610041 Sichuan Province People’s Republic of China

**Keywords:** Cancer, Computational biology and bioinformatics, Immunology

## Abstract

*SLC7A11* has significant translational value in cancer treatment. However, there are few studies on whether *SLC7A11* affects the immune status of lung adenocarcinoma (LUAD). Information on *SLC7A11* expression and its impact on prognosis was obtained from the cancer genome atlas and gene expression omnibus databases. The differentially expressed genes (DEGs) were analysed by GO and KEGG. GSEA enrichment analysis was performed in the *SLC7A11*-high and *SLC7A11*-low groups. The relationship between *SLC7A11* and tumour immunity, immune checkpoints, and immune cell infiltration was studied using R language. We analysed the correlation between *SLC7A11* and chemotactic factors (CFs) and chemokine receptors using the TISIDB database. *SLC7A11* is overexpressed in many tumours, including LUAD. The 5-year overall survival of patients in the *SLC7A11*-high group was lower than in the *SLC7A11*-low group. KEGG analysis found that the DEGs were enriched in ferroptosis signaling pathways. GSEA analysis found that the survival-related signaling pathways were enriched in the *SLC7A11*-low group. The *SLC7A11*-low group had higher immune scores and immune checkpoint expression. *SLC7A11* was negatively correlated with many immune cells (CD8+ T cells, immature dendritic cells), CFs, chemokine receptors (such as CCL17/19/22/23, CXCL9/10/11/14, CCR4/6, CX3CR1, CXCR3) and MHCs (major histocompatibility complex). *SLC7A11* may regulate tumour immunity and could be a potential therapeutic target for LUAD.

## Introduction

In recent years, with the accelerated development of targeted therapy and immunotherapy, the mortality rate of lung cancer has dramatically decreased. However, lung cancer remains the leading cause of cancer death in men and women over 50 years old^1^. Tumour resistance and immune escape limit the therapeutic effects in patients with advanced adenocarcinoma^2^. Therefore, finding new targets to prevent or slow disease progression is a priority.

SLC7A11 and SLC3A2 encode the cystine/glutamate transporter system (system x_c_^-^), which transports cysteine into cells in exchange for glutamate^3^. Both cysteine and glutamate are important endogenous substances. Cysteine is an important part of the redox system, playing a crucial role in balancing reactive oxygen species (ROS). ROS and ROS-mediated signaling pathways are involved in various cellular and biochemical processes, including cell apoptosis, ferroptosis, autophagy, cell proliferation and migration, endoplasmic reticulum stress, mitochondrial dysfunction, DNA damage, cell metabolism, and drug resistance^4^. Recent studies have found that *SLC7A11* may be an independent prognostic factor for LUAD^5^. However, whether *SLC7A11* can be an effective target for LUAD treatment requires more theoretical support.

In this study, we analysed the expression of *SLC7A11* and clinical information in LUAD patients. Then, we used GO/KEGG and GSEA for enrichment analysis. We analysed the correlation between *SLC7A11* and immune cells, checkpoint expression, and CFs.

The results showed that *SLC7A11* overexpression was negatively correlated with CFs in the immune microenvironment. Reduction of CFs might reduce immune cell infiltration, leading to immunosuppression and immune escape and thus protecting tumour cells from immune cell attack. Targeted inhibition of *SLC7A11* may enhance the tumour immune response.

## Materials and methods

### Data source

The TCGA database (https://portal.gdc.cancer.gov/) is an accessible data portal for collecting information related to cancer patients.

The TCGA database includes 54 normal samples and 497 LUAD samples. After removing samples with incomplete survival data and duplicates, a total of 468 LUAD samples with RNA-Seq expression and matched clinical information (including pathological staging, OS time, gender, and age) were obtained. The RNA-Seq expression data of a pan-cancer analysis were also obtained from the TCGA database. The GSE30219, GSE37745, and GSE68465 datasets were obtained from the GEO database (https://www.ncbi.nlm.nih.gov/geo/), and detailed information is shown in Table 1.

**Table 1:** The detailed information of three data sets in the GEO database.

Dataset	Platform	Submission date	Last update date	Country	Control	LUAD
GSE30219	GPL570	40,720	43,549	France	14	85
GSE37745	GPL570	41,032	44,707	Sweden	0	106
GSE68465	GPL96	42,125	43,322	USA	19	443

### DEGs analysis

We analysed differences in the expression of *SLC7A11* between normal and LUAD samples in the TCGA-LUAD、GSE30219, GSE37745, and GSE68465 datasets. We divided TCGA-LUAD samples into an *SLC7A11*-high group and *SLC7A11*-low group based on the mean expression value of *SLC7A11*. The DEGs between the *SLC7A11*-high and *SLC7A11*-low group were identified using the “limma” R package^6^ and a total of 622 DEGs were obtained (criteria: │logFC│ > 2 and adj.*P*.val < 0.05).

### Survival analysis

The “survival” and “survminer” R packages were used to perform survival analysis in the *SLC7A11*-high and *SLC7A11*-low groups in the TCGA-LUAD dataset. We validated the survival analysis results in the GSE68465 dataset.

We also performed a survival analysis in the KM Plotter database (https://kmplot.com/analysis/). In the lung cancer analysis interface, we selected “adenocarcinoma” as the “histology, “OS” for “survival,” “60 months” as the “follow-up threshold”, “*SLC7A11*” as the “gene symbol”, and “auto selected best cutoff” for “split patients by”. A total of 1161 LUAD samples were analysed for survival. In the pan-cancer analysis interface, 7462 pan-cancer samples were analysed for survival, and detailed information on the pan-cancer samples is shown in Table 2.

**Table 2:** The detailed information of pan-cancer survival analysis in KM-plot.

Cancer	Number	*p*
Bladder carcinoma	405	0.036
Breast cancer	1090	0.0109
Cervical squamous cell carcinoma	304	0.0085
Esophageal adenocarcinoma	80	0.079
Esophageal squamous cell carcinoma	81	0.11
Head-neck squamous cell carcinoma	500	0.0037
Kidney renal clear cell carcinoma	530	0.00058
Kidney renal papillary cell carcinoma	288	4.9e-10
Liver hepatocellular carcinoma	371	8.2e-8
Lung adenocarcinoma	513	0.0042
Ovarian cancer	374	0.00055
Pancreatic ductal adenocarcinoma	177	0.01
Pheochromocytoma and Paraganglioma	178	0.067
Rectum adenocarcinoma	165	0.0062
Sarcoma	259	0.011
Stomach adenocarcinoma	375	0.071
Testicular germ cell tumor	134	0.13
Thymoma	119	0.026
Thyroid carcinoma	502	0.062
Uterine corpus endometrial carcinoma	543	0.0043

### Gene enrichment analysis

GO analysis includes three parts: molecular function (MF), biological process (BP), and cellular component (CC)^7^. The KEGG pathway database is currently the most widely used public database of metabolic pathways^8^. We used the Database for Annotation, Visualization and Integrated Discovery (DAVID) (https://david.ncifcrf.gov/tools.jsp) to perform GO and KEGG analysis on the 622 DGEs.

GSAE analysis was performed using “c2.cp.kegg.v7.4.symbols.gmt” as the reference genome in R language^9^.

### The relationship between *SLC7A11* and tumour immunity

In the TCGA-LUAD dataset, the “commonGenes.gct” and “estimateScore.gct” files were read in R language, and the "estimate" was used to score the immune score, stromal score, and immune cell infiltration for each tumour sample^10^. We compared the immune and stromal scores between the *SLC7A11*-high and *SLC7A11*-low group using the “limma” package, and analysed the correlation between *SLC7A11* and immune cell infiltration and common immune checkpoints using the “corrplot” package. The correlation between *SLC7A11* and *SLC3A2* was analyzed by the Sangbox 3.0 (http://sangerbox.com/home.html).

We validated the correlation between *SLC7A11* and immune cell infiltration in the GSE30219, GSE37745, and GSE68465 datasets.

### Relationship between *SLC7A11* and CFs

We evaluated the correlation between *SLC7A11* and CFs, chemokine receptors, and MHCs in the tumour microenvironment using the “chemokines” module of the TISIDB database (http://cis.hku.hk/TISIDB/).

### Screening of potential therapeutic drugs

We then used the “pRRophetic” R package to assess the treatment response, as indicated by the IC_50_ of targeted and immunotherapy drugs^11^.

## Result

### Overexpression of *SLC7A11* in LUAD

**Figure 1:**
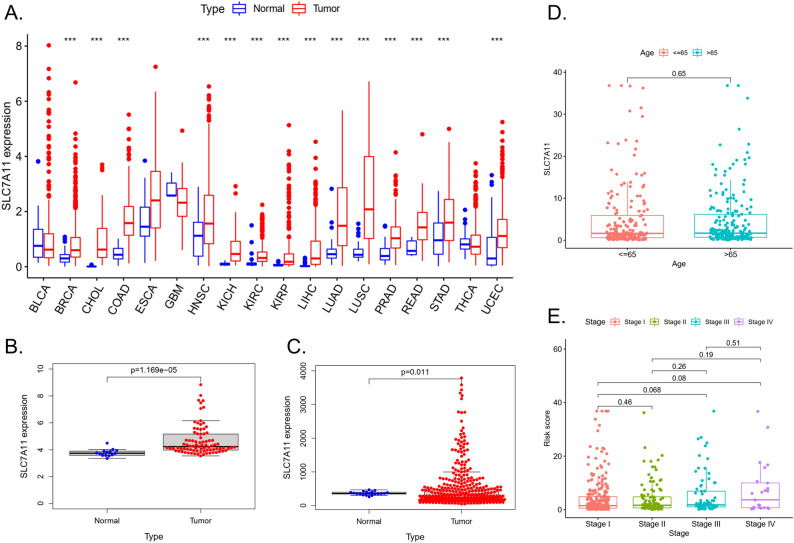
The expression of *SLC7A11* in the tumor. (**A**) *SLC7A11* expression in distinct cancers compared with normal tissues in TCGA. (**B**) *SLC7A11* expression LUAD compared with normal tissues in the GSE30219 database. (**C**) *SLC7A11* expression LUAD compared with normal tissues in the GSE68465 database. (**D**) *SLC7A11* expression of different ages in LUAD patients. E. *SLC7A11* expression of different stages in LUAD patients.

In the pan-cancer analysis, *SLC7A11* was found to be overexpressed in tumours such as breast cancer (BRCA), cholangiocarcinoma (CHOL), colon adenocarcinoma (COAD), head and neck squamous cell carcinoma (HNSC), kidney renal clear cell carcinoma (KIRC), kidney renal papillary cell carcinoma (KIRP), liver hepatocellular carcinoma (LIHC), lung squamous cell carcinoma (LUSC), LUAD, prostate adenocarcinoma (PRAD), rectum adenocarcinoma (READ), stomach adenocarcinoma (STAD), and uterine corpus endometrial carcinoma (UCEC) (Fig. 1A).

In the GSE30219 and GSE68465 datasets, the expression of *SLC7A11* was higher in LUAD than in normal tissues (*p* < 0.05) (Fig. 1B,C).

The TCGA-LUAD dataset showed no significant difference in *SLC7A11* expression among ages or stages (Fig. 1D,E).

### Relationship between *SLC7A11* and prognosis in LUAD

**Figure 2:**
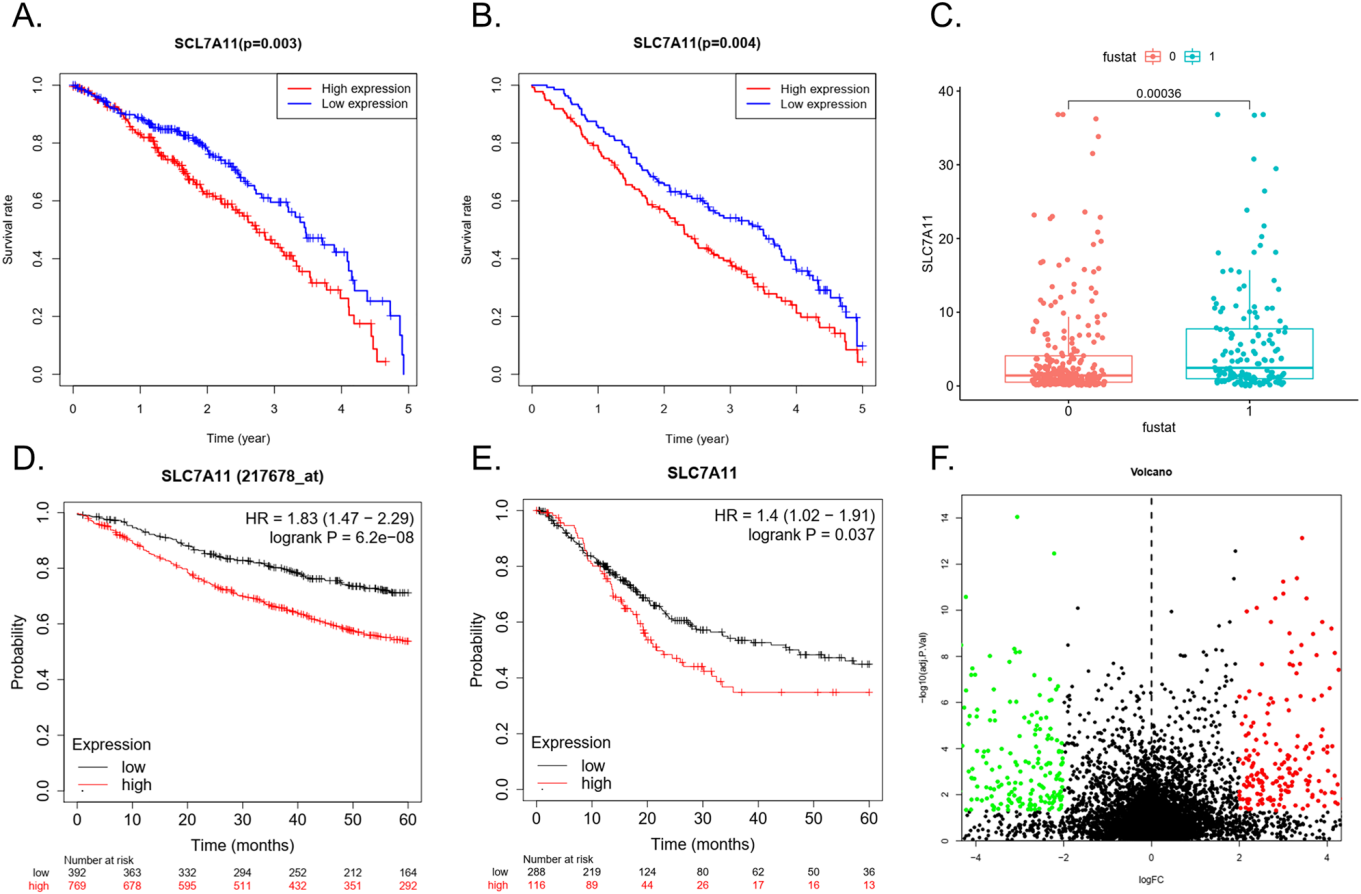
Survival analysis. (**A**) 5-year OS of the *SLC7A11*-high group and *SLC7A11*-low group in TCGA-LUAD dataset. (**B**) 5-year OS of the *SLC7A11*-high group and *SLC7A11*-low group in the GSE68465 dataset. (**C**) The expression of *SLC7A11* in LUAD with different survival states in the TCGA-LUAD dataset. Survival curve of LUAD in KM-plot. Survival curve of pan-cancer in KM-plot (Bladder Carcinoma). (**F**). DEGs in the *SLC7A11*-high group and *SLC7A11*-low group.

Survival analysis of the TCGA-LUAD dataset showed that the 5-year OS of the *SLC7A11*-high group was lower than that of the *SLC7A11*-low group (*p* < 0.05) (Fig. 2A). In the GSE68465 dataset the *SLC7A11*-low group had a longer 5-year OS than the *SLC7A11*-high group. (*p* < 0.05) (Fig. 2B). The expression of *SLC7A11* was higher in deceased patients than in surviving patients (Fig. 2C).

In the Kaplan–Meier Plotter database, the *SLC7A11*-low group showed a longer 5-year OS than the *SLC7A11*-high group (*p* < 0.05) (Fig. 2D). The pan-cancer analysis showed that the 5-year OS of the *SLC7A11*-high group was lower than that of the *SLC7A11*-low group for many tumours (*p* < 0.05) (Fig. 2E and Table 2).

### DEGs analysis and GO/KEGG analysis

The TCGA-LUAD database contained 622 DEGs between the *SLC7A11*-high and *SLC7A11*-low groups (Fig. 2F).

**Figure 3:**
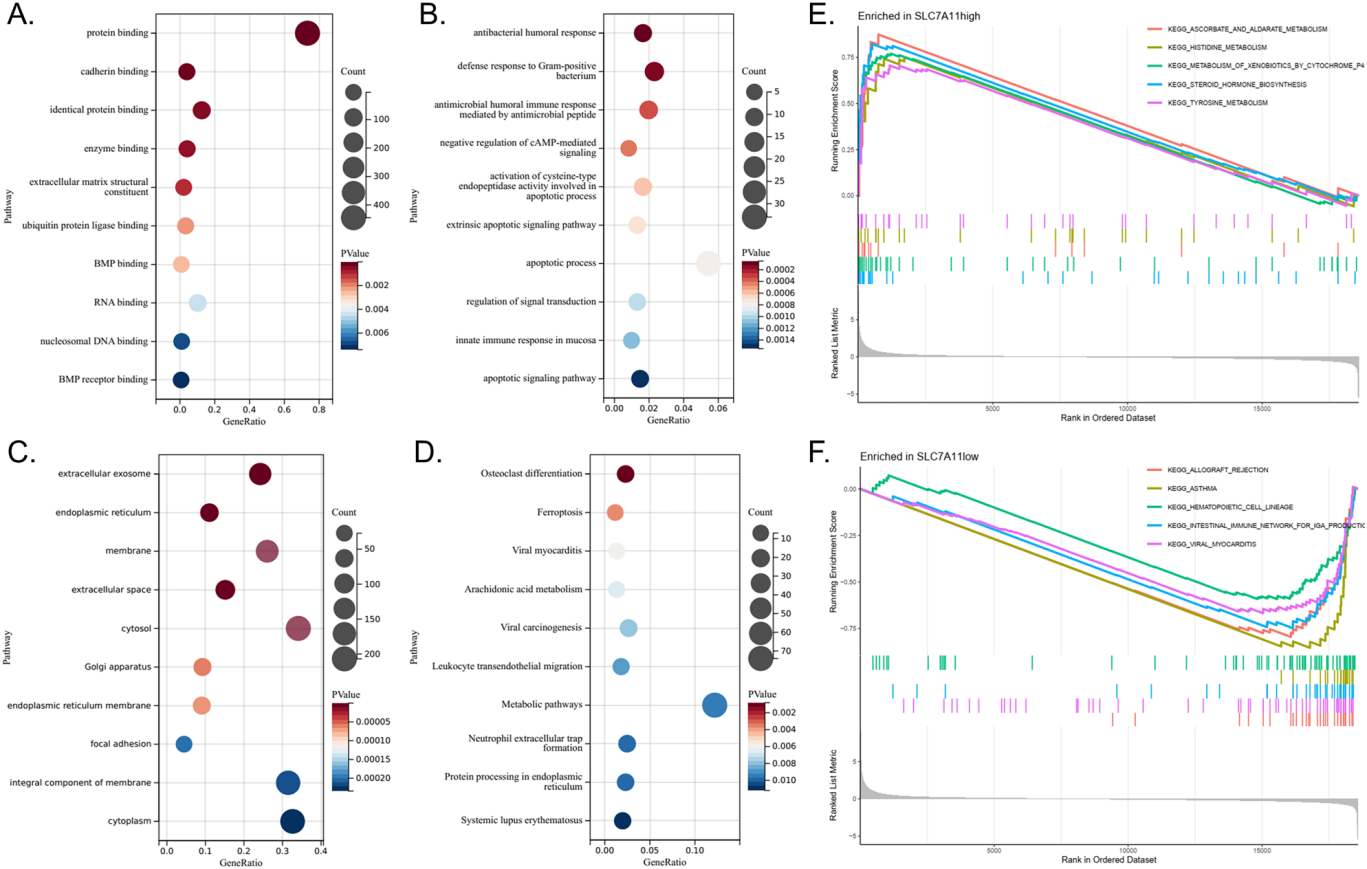
Enrichment analysis. (**A**) GO-MF analysis of DEGs in LUAD. (**B**) GO-BP analysis of DEGs in LUAD. (**C**) GO-CC analysis of DEGs in LUAD. (**D**) GO-KEGG analysis of DEGs in LUAD. (**E**) GSEA analysis in the *SLC7A11*-high group. (**F**) GSEA analysis in the *SLC7A11*-low group.

GO-MF analysis showed that DEGs are mainly enriched in molecular function pathways such as protein binding, cadherin binding, identical protein binding, and enzyme binding (Fig. 3A).

GO-BP analysis showed that the DEGs were mainly enriched in the defense response to Gram-positive bacteria, the negative regulation of cAMP-mediated signaling, and apoptosis-related biological processes. More details are shown in Fig. 3B.

GO-CC analysis showed that DEGs were mainly enriched in cellular components such as extracellular exosomes, endoplasmic reticulum, membranes, extracellular spaces, and cytosol (Fig. 3C).

KEGG enrichment analysis found that DEGs were mainly enriched in pathways such as osteoclast differentiation, ferroptosis, viral myocarditis, arachidonic acid metabolism, and viral carcinogenesis, among which four pathways were related to immunity, three pathways were related to metabolism, and one pathway was related to ferroptosis (Fig. 3D).

GSEA analysis revealed that in the *SLC7A11*-high group, gene sets were mainly enriched in metabolism-related signaling pathways, such as steroid hormone biosynthesis, metabolism of xenobiotics by cytochrome p450, ascorbate and aldarate metabolism, histidine metabolism, and tyrosine metabolism (Fig. 3E).

In the *SLC7A11*-low group, gene sets were mainly enriched in immune-related signaling pathways, such as viral myocarditis, the intestinal immune network for IgA production, asthma, and allograft rejection (Fig. 3F).

### The relationship between *SLC7A11* and tumour immunity

KEGG and GSEA analysis showed differences in immune-related signaling pathways between the *SLC7A11*-high group and *SLC7A11*-low group. Therefore, we also analysed the immune status of the two groups.

**Figure 4:**
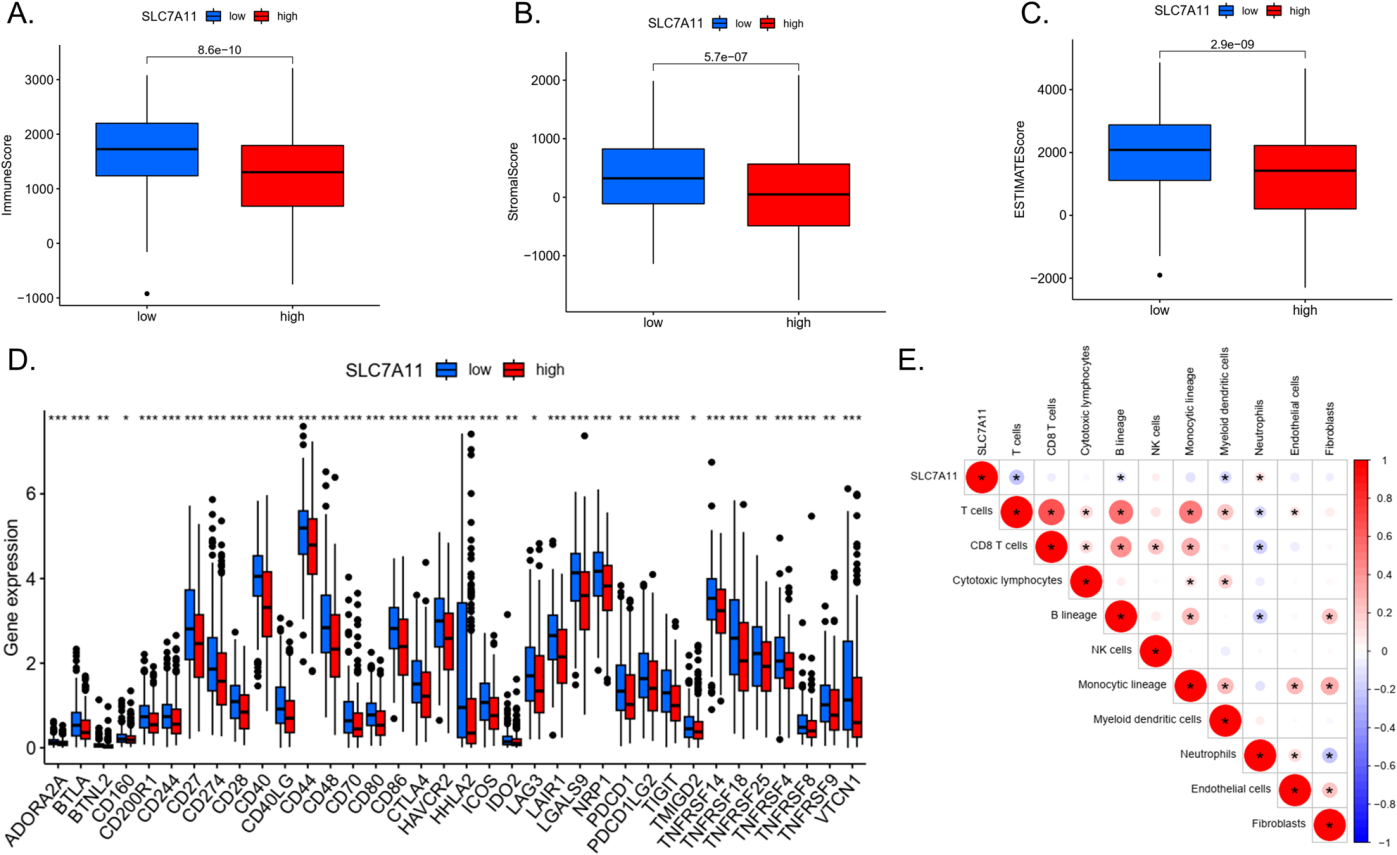
Immune analysis. (**A**) Immune cells score in the *SLC7A11*-high group and *SLC7A11*-low group. (**B**) Stromal cells score in the *SLC7A11*-high group and *SLC7A11*-low group. (**C**) ESTIMATE scores in the *SLC7A11*-high group and *SLC7A11*-low group. (**D**) The difference of 29 immune checkpoints expression in the *SLC7A11*-high group and *SLC7A11*-low group. (**E**) The correlation between *SLC7A11* expression and immune cells in the TCGA-LUAD database. (***p < 0.001; **p < 0.01; *p < 0.05).

In the TCGA-LUAD dataset, the immune (Fig. 4A), stromal (Fig. 4B), and ESTIMATE (Fig. 4C) scores of the *SLC7A11*-high group were lower than those of the *SLC7A11*-low group. *SLC7A11* expression was negatively correlated with many immune checkpoints, such as *BTLA*, *CD244*, *CD247*, *CD40*, *CTLA4*, and *ICOS* (Fig. 4D).

**Figure 5:**
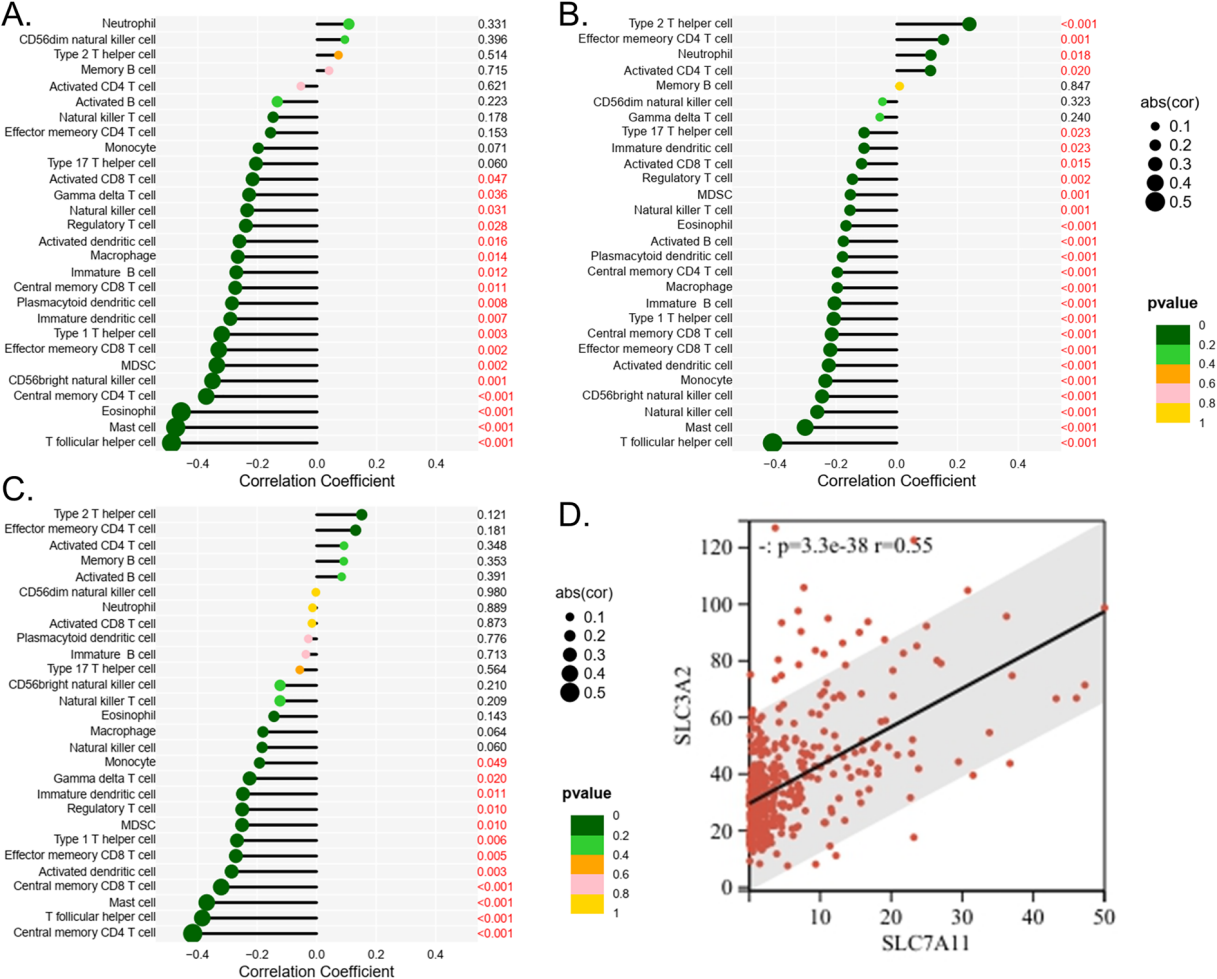
(**A**) The correlation between *SLC7A11* expression and immune cells in the GSE30219 database. (**B**) The correlation between *SLC7A11* expression and immune cells in the GSE37745 database. (**C**) The correlation between *SLC7A11* expression and immune cells in the GSE68465 database. (**D**) The correlation between *SLC7A11* expression and SLC3A2 in the TCGA-LUAD database.

*SLC7A11* expression was negatively correlated with T cells, B lineage, and myeloid dendritic cells, but positively correlated with neutrophils (Fig. 4E). In the GSE37745, GSE68465, and GSE68465 datasets, the expression of *SLC7A11* was negatively correlated with many immune cells, such as central memory CD8 T cells, effector memory CD8 T cells, immature dendritic cells, and type 1 T helper cells. In the GSE68465 dataset, *SLC7A11* was also positively correlated with neutrophils (Fig. 5A,B,C).

### Relationship between *SLC7A11* and CFs

The role of CFs in tumour immunity is related to tumour immune escape. *SLC7A11* expression was negatively correlated with most CFs (Table 3), chemokine receptors (Table 4), and MHCs (Table 5).

**Table 3:** The correlation between *SLC7A11* expression and CFs.

Chemokines	Rho	*p*	Chemokines	Rho	*p*
*XCL2*	− 0.078	0.0753	*CCL5*	− 0.106	0.0162
*XCL1*	− 0.042	0.3410	*CCL4*	− 0.091	0.0386
*CXCL9*	− 0.103	0.0196	*CCL3*	− 0.103	0.0187
*CXCL8*	0.106	0.0163	*CCL28*	− 0.350	0.0000
*CXCL6*	− 0.060	0.1740	*CCL26*	0.060	0.1730
*CXCL5*	0.092	0.0372	*CCL24*	0.006	0.8860
*CXCL3*	0.034	0.4380	*CCL23*	− 0.222	0.0000
*CXCL2*	− 0.038	0.3940	*CCL22*	− 0.224	0.0000
*CXCL17*	− 0.124	0.0047	*CCL21*	− 0.014	0.7450
*CXCL16*	− 0.055	0.2090	*CCL20*	0.172	0.0001
*CXCL14*	− 0.191	0.0000	*CCL2*	− 0.175	0.0001
*CXCL13*	− 0.105	0.0173	*CCL19*	− 0.241	0.0000
*CXCL12*	− 0.191	0.0000	*CCL18*	− 0.040	0.3620
*CXCL11*	− 0.125	0.0045	*CCL17*	− 0.269	0.0000
*CXCL10*	− 0.118	0.0072	*CCL15*	0.137	0.0018
*CXCL1*	− 0.012	0.7910	*CCL14*	− 0.144	0.0010
*CX3CL1*	− 0.434	0.0000	*CCL13*	− 0.210	0.0000
*CCL8*	− 0.087	0.0471	*CCL11*	− 0.160	0.0003
*CCL7*	− 0.084	0.0577

**Table4:** The correlation between *SLC7A11* expression and chemokine receptor.

Receptors	Rho	*p*	Receptors	Rho	*p*
*CCR1*	− 0.118	0.0071	*CCR10*	− 0.045	0.3090
*CCR2*	− 0.304	0.0000	*CX3CR1*	− 0.281	0.0000
*CCR3*	0.151	0.0006	*CXCR1*	0.137	0.0019
*CCR4*	− 0.253	0.0000	*CXCR2*	− 0.053	0.2250
*CCR5*	− 0.222	0.0000	*CXCR3*	− 0.253	0.0000
*CCR6*	− 0.268	0.0000	*CXCR4*	− 0.184	0.0000
*CCR7*	− 0.236	0.0000	*CXCR5*	− 0.18	0.0000
*CCR8*	− 0.181	0.0000	*CXCR6*	− 0.145	0.0010

**Table 5:** The correlation between *SLC7A11* expression and chemokine *MHCs*.

Receptors	Rho	*p*	Receptors	Rho	*p*
*B2M*	− 0.088	0.0449	*HLA-DQA2*	− 0.301	3.33E-12
*HLA-A*	− 0.098	0.026	*HLA-DQB1*	− 0.343	1.22E-15
*HLA-B*	− 0.224	2.76E-07	*HLA-DRA*	− 0.381	< 2.2e-16
*HLA-C*	− 0.144	0.00107	*HLA-DRB1*	− 0.367	1.67E-19
*HLA-DMA*	− 0.356	5.63E-17	*HLA-E*	− 0.208	2.01E-06
*HLA-DMB*	− 0.355	7.47E-17	*HLA-F*	− 0.165	0.000165
*HLA-DOA*	− 0.309	8.83E-13	*HLA-G*	− 0.114	0.00981
*HLA-DOB*	− 0.244	2.05E-08	*TPA1*	− 0.046	0.292
*HLA-DPA1*	− 0.386	< 2.2e-16	*TPA2*	− 0.044	0.319
*HLA-DPB1*	− 0.378	< 2.2e-16	*TAPBP*	− 0.031	0.483
*HLA-DQA1*	− 0.281	8.76E-11			

### Relationship between *SLC7A11* and *SLC3A2*

In the TCGA-LUAD dataset, the expression of *SLC7A11* showed a significant correlation with that of *SLC3A2* (*p* < 0.05) (Fig. 5D), a known partner for *SLC7A11*. In the TCGA-LUAD dataset, the immune (Supplement Fig. 1A), stromal (Supplement Fig. 1B), and ESTIMATE (Supplement Fig. 1C) scores of the *SLC3A2*-high group were lower than those of the *SLC3A2*-low group.

### Drug sensitivity analysis

**Figure 6:**
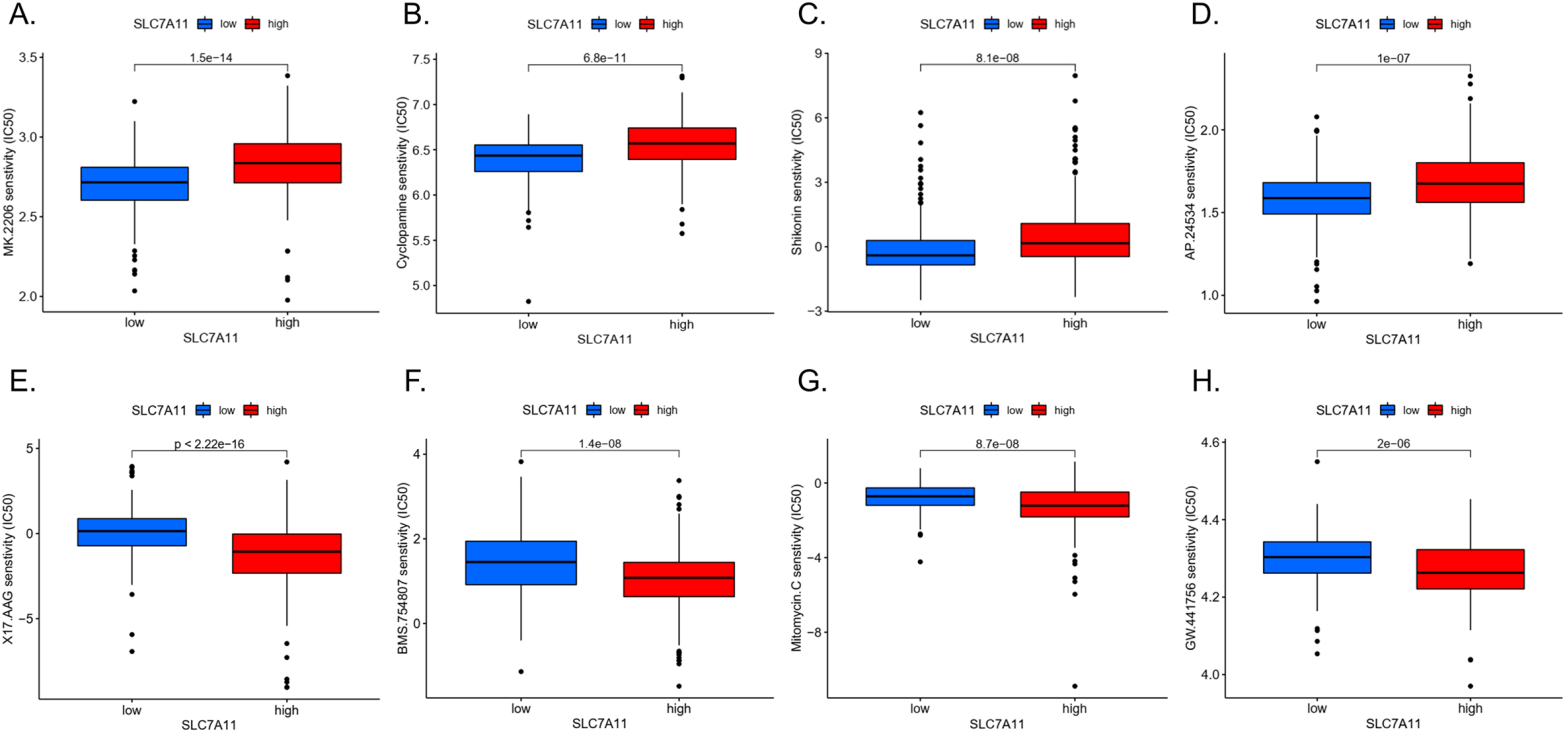
Drug sensitivity analysis. (**A**)–(**D**) showed that the drug had a lower IC_50_ value in the *SLC7A11*-high group. (**E**)–(**H**) showed that the drug had a lower IC_50_ value in the *SLC7A11*-low group.

We identified 10 immunotherapeutic or targeted agents that showed lower IC_50_ values in the *SLC7A11*-high group than in the *SLC7A11*-low group, including two insulin-like growth factor 1 receptor/insulin receptor inhibitors, two HSP90 ATPase inhibitors, and two tyrosine, serine/threonine kinase inhibitors (Fig. 6A,B,C,D). We also identified 20 drugs with low IC_50_ values in the *SLC7A11*-low group (Fig. 6E,F,G,H).

## Discussion

With the development of early detection, surgical treatment, precise pathological staging, targeted therapy and immunotherapy, the survival rate of lung cancer has improved. The 3-year OS for lung cancer increased from 22% in 2004–2006 to 33% in 2016–2018. However, lung cancer remains the leading cause of cancer death in men and women aged 50 and over^1^. Among all lung cancers, LUAD accounts for about 50% of cases.

This study found that *SLC7A11* is highly expressed in cancers such as breast cancer, LUSC, LUAD, and gastric cancer. In addition, patients with high *SLC7A11* expression had a lower 5-year OS than those with low *SLC7A11* expression.

Treatment failure in LUAD is related to many factors, such as tumour heterogeneity, immune escape, and chemotherapy resistance^12^. Most studies suggest that *SLC7A11* overexpression is related to immune escape. The current study found that high *SLC7A11* expression is associated with treatment failure in immune therapy, indicating that *SLC7A11* is associated with the efficacy of immune therapy. This is because high *SLC7A11* expression can increase the concentration of specific metabolites in tumour cells, such as GSH, which inhibits the activation of immune cells and the apoptosis of tumour cells, thereby reducing the efficacy of immune therapy^13^. Some studies have shown that *SLC7A11* can affect tumour immune escape and the anti-tumour immune response by regulating intracellular redox balance and tumour cell metabolism and growth. Inhibiting *SLC7A11* can enhance the attack of tumour cells by immune cells and improve the efficacy of immune therapy^14^. However, blocking the expression of *SLC7A11* does not affect the normal development of mouse embryos^15^. Therefore, drugs that block *SLC7A11* may be ideal anticancer drugs.

Ferroptosis is a newly discovered type new type of cell death caused by the excessive accumulation of iron ions in cells. It was found that *SLC7A11* plays an essential protective role in ferroptosis. Ferroptosis may exert antitumour effects by boosting the immune response and killing cancer cells, and it also has a direct impact on immune cells^16^. KEGG analysis found that the DEGs were enriched in the ferroptosis signaling pathway.

In this study, we found that as well as a lower immune score, matrix score, and ESTIMATE score in the *SLC7A11*-high group, expression of the immune checkpoint, including *CD274* (*PD-L1*), *CTLA4*, and *LAG3*, was also lower. GSEA analysis showed that signaling pathways related to metabolism were enriched in the *SLC7A11*-high group, while signaling pathways related to immunity were enriched in the *SLC7A11*-low group. In TCGA-LUAD, *SLC7A11* was negatively correlated with the expression of T cells, B lymphocytes, and myeloid cells and positively correlated with the expression of neutrophils.

A high proportion of neutrophils can inhibit the function of immune cells such as CD8+ T cells and promote tumour growth^17^. In the GEO dataset, the expression of *SLC7A11* was negatively correlated with the central memory CD8 T cells, effector memory CD8 T cells, immature dendritic cells, and type 1 T helper cells, all of which play an important role in killing tumour cells. In the GSE68465 dataset, we also observed a positive correlation between *SLC7A11* and neutrophils, which is consistent with TCGA-LUAD.

Interestingly, the expression of *SLC7A11* was negatively correlated with the expression of Treg (regulatory T cell) and MDSC (myeloid-derived suppressor cells). Treg cells and MDSC play an important role in the immune escape of tumour cells in the tumour microenvironment^18^. Therefore, the impact of *SLC7A11* on tumour immune cells may be very complex.

Activated CD8+ T cells release IFN-γ and then activate the JAK-STAT1 pathway, resulting in the translocation of phosphorylated STAT1 to the nucleus, thus inhibiting the transcription of *SLC7A11*^19,20,21,22,23^. In addition, *TGF-β1* (transforming growth factor β1) released by macrophages can inhibit the expression of *SLC7A11* by activating the SMAD-dependent signaling pathway and finally lead to ferroptosis of cancer cells^24^. Therefore, inhibiting *SLC7A11* may be a promising target for tumour immune therapy.

CFs are part of the cytokine family and play an essential role in the tumour microenvironment. CFs can attract immune cells to enter the tumour microenvironment, thereby affecting the response of the tumour to immunotherapy^25^. Tumours with fewer CHs may be unresponsive to immune therapy, leading to tumour immune escape.

This study confirmed that the expression of *SLC7A11* was negatively correlated with the expression of *CCL17/19/22/23*, *CXCL9/10/11/14*, *CCR4/6*, *CX3CR1*, and *CXCR3*, suggesting that high *SLC7A11* expression may inhibit the migration of immune cells. Studies have shown that *CXCL9/10/11* are the main CFs for CD8+ T cells^26,27,28^, and that *CXCL9/10* can attract Th1 cells to the tumour microenvironment and play an important role against cancer^28,27^. *CCL17* is the ligand of *CCR4*, and they affect the recruitment of Treg and Th17 cells in tumours^29^. *CXCR3* and *CX3CR1* are mainly responsible for infiltrating tumour-inhibitory lymphocytes into the tumour microenvironment^30,31^, further confirming the results of immune cell infiltration. This may explain why patients with high *SLC7A11* expression have poor responses to immunotherapy. The chemokine *CXCL14* is a key regulatory factor in cancer and a potential target for future cancer immunotherapy^32^. There is also evidence that high *SLC7A11* expression can affect the expression of immune inhibitors and immune stimulators. Therefore, these results suggest that *SLC7A11* may play a role in regulating tumour immunity.

MHCs are a group of highly polymorphic genes, and proteins encoded by MHCs play an important role in the immune system. MHCs are divided into two categories: MHC-I and MHC-II. MHC-I and MHC-II interact with CD8+ T cells and CD4+ T cells respectively, thus activating the immune response^33,34^. Tumour cells usually express abnormal MHC-I molecules, which allows them to escape attack by CD8+ T cells. In addition, tumour cells can escape immune surveillance by reducing MHC-I expression^35^. Therefore, restoring MHC-I expression is very important for tumour immunotherapy^36^. MHC-II can also affect tumour immunity by regulating the activity of immune cells in the tumour microenvironment^37^.

Previous studies have shown that *SLC7A11* is correlated with *SLC3A2*^38^. Our study found that the expression of *SLC3A2* was related to *SLC7A11*. In LUAD, we found a lower immune score, matrix score, and ESTIMATE score in the *SLC3A2*-high group, which is consistent with the immune status of the *SLC7A11*-high group. SLC3A2 is the chaperone protein of SLC7A11, which is used to maintain the stability of SLC7A11 protein and regulate the transport of SLC7A11 to the plasma membrane. In system Xc-, SLC7A11 plays a significant role in transport function^39^.

*SLC7A11* immune-targeted agents may also be effective adjuvants for chemotherapy. Conti et al. studied the use of combination immunotherapy against HER2 and *SLC7A11*. Antibodies targeting HER2 and SLC7A11 can inhibit the proliferation and metastasis of BCSCs, exerting synergistic or complementary effects^40^. All of the above suggest that *SLC7A11* may be an important target for treating LUAD, possibly via the regulation of tumour immunity by *SLC7A11*.

Although our research has limitations, it can be considered a verification that we use multiple database sets for analysis. In addition, we used many methods and aspects to verify the relationship between *SLC7A11* and tumor immunity. In order to further verify this exciting discovery, we have been purchasing LUAD cell lines and drugs and collecting clinical specimens of LUAD patients for the next experiment.

## Conclusion

In conclusion, this study found that *SLC7A11* was significantly upregulated and may be associated with the prognosis of LUAD. The expression of *SLC7A11* is negatively correlated with the expression of immune checkpoints *PD-L1*, *CTLA4*, and et al. *SLC7A11* is negatively correlated with the expression of many CFs, leading to the loss of immune cells such as CD8+ T cells and causing immune suppression. Regulating the expression of *SLC7A11* may be a potential target for tumor immunotherapy.

### Supplementary Information

Supplementary Figure 1.

Supplementary Information 2.

## Data Availability

The TCGA-LUAD dataset was downloaded from the TCGA database (https://portal.gdc.cancer.gov/). The GSE30219 dataset was downloaded from the GEO database (https://www.ncbi.nlm.nih.gov/geo/query/acc.cgi?acc=GSE30219). The GSE37745 dataset was downloaded from the GEO database (https://www.ncbi.nlm.nih.gov/geo/query/acc.cgi?acc=GSE37745). The GSE68465 dataset was downloaded from the GEO database (https://www.ncbi.nlm.nih.gov/geo/query/acc.cgi?acc=GSE68465).

## Abbreviations

BP: Biological process
BRCA: Breast cancer
CC: Cellular component
*CCL*: C–C motif chemokine ligand
*CCR*: C–C motif chemokine receptor
CFs: Chemotactic factors
CHOL: Cholangio carcinoma
COAD: Colon adenocarcinoma
*CTLA4*: Cytotoxic T-Lymphocyte associated Antigen 4
*CX3CR*: C-X3-C motif chemokine receptor
*CXCL*: C-X-C motif chemokine ligand
DEGs: Differentially expressed genes
GEO: Gene expression omnibus
GO: Gene ontology
GSEA: Gene set enrichment analysis
GSH: Glutathione
HNSC: Head and neck squamous cell carcinoma
KEGG: Kyoto encyclopedia of genes and genomes
KIRC: Kidney renal clear cell carcinoma
KIRP: Kidney renal papillary cell carcinom
*LAG3*: Lymphocyte activating 3
LIHC: Liver hepatocellular carcinoma
LUAD: Lung adenocarcinoma
LUSC: Lung squamous cell carcinoma
MDSC: Myeloid-derived suppressor cells
MF: Molecular function
MHC: Major histocompatibility complex
MHCs: Major histocompatibility complex
NSCLC: Non-small cell lung cancer
OS: Overall survival
*PD-L1*: Programmed death ligand 1
PRAD: Prostate adenocarcinoma
READ: Rectum adenocarcinoma
ROS: Reactive oxygen species
*SLC7A11*: Solute carrier family 7 member 11
STAD: Stomach adenocarcinoma
TCGA: The cancer genome atlas
*TGF-β1*: Transforming growth factor β1
Treg: Regulatory T cell
x_c_-: The cystine/glutamate transporter system
UCEC: Uterine corpus endometrial carcinoma
