# Supplementary figures and images for: SLC7A11, a potential immunotherapeutic target in lung adenocarcinoma
Source: Sci Rep. 2023 Oct 25;13:18302. doi: 10.1038/s41598-023-45284-z (PMC10600206; doi:10.1038/s41598-023-45284-z)

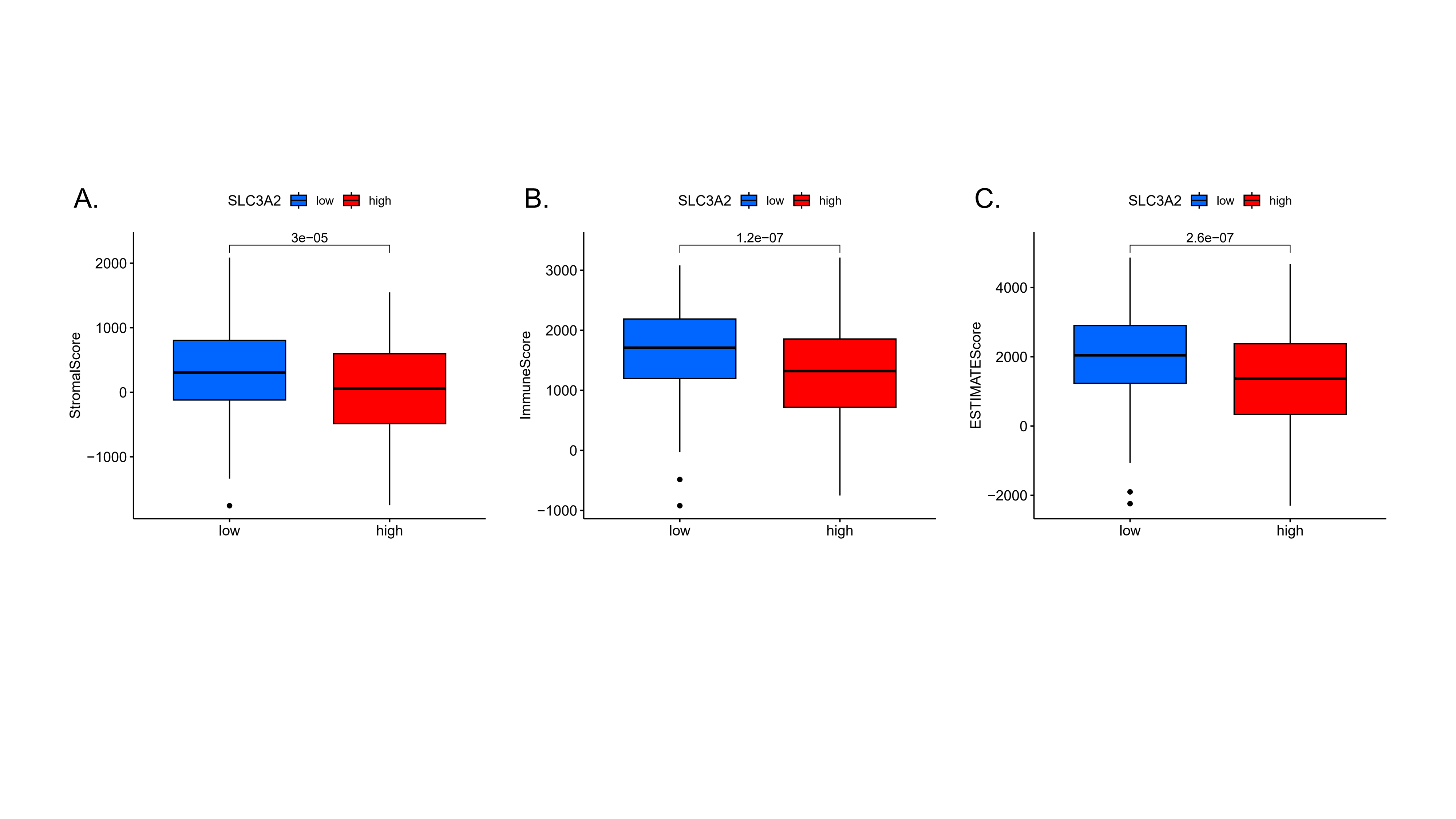

Supplement: Supplementary file 1 — Supplementary Figure 1. [file 41598_2023_45284_MOESM1_ESM.tif]
