# Supplementary material for: SLC7A11, a potential immunotherapeutic target in lung adenocarcinoma
Source: Sci Rep. 2023 Oct 25;13:18302. doi: 10.1038/s41598-023-45284-z (PMC10600206; doi:10.1038/s41598-023-45284-z)
Supplement: Supplementary file 2 — Supplementary Information 2. [file 41598_2023_45284_MOESM2_ESM.docx]

**Supplement Figure 1.** A. Immune cells score in the *SLC3A2*-high group and *SLC3A2*-low group. B. Stromal cells score in the *SLC3A2*-high group and *SLC3A2*-low group. C. STIMATE scores in the *SLC3A2*-high group and *SLC3A2*-low group.
